# Titrating Growth Hormone Dose to High-Normal IGF-1 Levels Has Beneficial Effects on Body Fat Distribution and Microcirculatory Function Despite Causing Insulin Resistance

**DOI:** 10.3389/fendo.2020.619173

**Published:** 2021-02-09

**Authors:** Christa C. van Bunderen, Rick I. Meijer, Paul Lips, Mark H. Kramer, Erik H. Serné, Madeleine L. Drent

**Affiliations:** ^1^ Section of Endocrinology, Neuroscience Campus Amsterdam, Department of Internal Medicine, Amsterdam UMC, Vrije Universiteit Amsterdam, Amsterdam, Netherlands; ^2^ Division of Endocrinology, Department of Internal Medicine, Radboud University Medical Center, Nijmegen, Netherlands; ^3^ Section of Vascular Medicine, Department of Internal Medicine, Amsterdam Cardiovascular Sciences, Amsterdam UMC, Vrije Universiteit Amsterdam, Amsterdam, Netherlands; ^4^ Department of Internal Medicine, Amsterdam UMC, Vrije Universiteit Amsterdam, Amsterdam, Netherlands

**Keywords:** growth hormone, insulin-like growth factor-1, growth hormone deficiency in adults, insulin resistance, vasomotion, vascular endothelium, growth hormone treatment

## Abstract

To clarify the mechanism underlying the described U-shaped relation of both low and high levels of IGF-1 with cardiovascular disease this study explores the effect of decreasing and increasing growth hormone dose in GH deficient adults on (micro)vascular function, body composition and insulin resistance. In this randomized clinical trial, thirty-two subjects receiving GH therapy with an IGF-1 concentration between −1 and 1 SD score (SDS) for at least one year were randomized to receive either a decrease (IGF-1 target level of −2 to −1 SDS) or an increase of their daily GH dose (IGF-1 target level of 1 to 2 SDS) for a period of 24 weeks. Microvascular endothelium (in)dependent vasodilatation and vasomotion, vascular stiffness by pulse wave analysis, and HOMA-IR were measured. At the end of the study 30 subjects (65.6% men, mean age 46.6 (SD 9.9) years) were analyzed. There was a favorable effect of increasing the IGF-1 level on waist circumference compared to decreasing the IGF-1 level (p=0.05), but a detrimental effect on insulin resistance (p=0.03). Decreasing IGF-1 level significantly lowered the endothelial domain of vasomotion (p=0.03), whereas increasing IGF-1 level increased the contribution of the neurogenic domain (p=0.05). This change was related to the favorable change in waist circumference. In conclusion, increasing IGF-1 levels was beneficial for body composition but detrimental with respect to insulin resistance. The contribution of the neurogenic vasomotion domain increased in parallel, and could be explained by the favorable change in waist circumference.

**Clinical Trial Registration:**

ClinicalTrials.gov, identifier NCT01877512.

## Introduction

Epidemiological evidence for a bidirectional link between serum IGF-1 concentrations and cardiovascular disease (CVD) has been repeatedly demonstrated. On the one hand, growth hormone (GH) deficient adults are characterized by an adverse lipid profile and altered body composition with increased fat mass which may put them on an increased risk for cardiovascular disease (1, 2). Moreover, even low-normal IGF-1 levels have been associated with the development of ischemic heart disease and stroke in the general population (3–5). On the other hand, high levels of IGF-1 such as observed in acromegaly are also associated with an adverse cardiovascular risk profile and a higher prevalence of CVD (6). These data suggest a U-shaped relationship between IGF-1 concentrations and CVD, which was corroborated by the finding of a U-shaped relationship with cardiovascular mortality in a Dutch cohort of healthy older people (7). Presently, it is unclear whether such a U-shaped association also exists in GH deficient adults treated with GH. Many studies demonstrate favorable effects of GH replacement therapy in adults with GH deficiency (8, 9), and of normalization of GH and IGF-1 levels in acromegaly (10), on cardiovascular risk factors, but the presented data suggest that there may be an optimal target level of IGF-1. In addition, the underlying mechanisms of this U-shaped relationship remain unresolved. On the one hand, IGF-1 is postulated to protect against (micro)vascular endothelial dysfunction, atherosclerotic plaque development, and ischemic myocardial damage (11). Interestingly, cultured endothelial cells, isolated microvessels, as well as the capillaries of perfused hearts, all possess distinct surface binding sites for both IGF-1 and insulin (12). Capillary density has been shown to be lower in untreated GH deficient patients than in control subjects, which increased to a level that was not different from that in control subjects after GH treatment normalized plasma IGF-1 (13). On the other hand, high levels of IGF-1 such as observed in acromegaly are associated with profound insulin resistance (6), which may offset the beneficial (micro)vascular effects of IGF-1. Insulin resistance itself has been linked to a lower capillary density and a change in vasomotion, the rhythmic change in vascular diameter, which is thought to influence capillary perfusion (14).

In order to elucidate possible mechanisms underlying the U-shaped relationship of IGF-1 with CVD, the aim of the present study is to explore the effects of titrating GH dose to low-normal or high-normal levels of IGF-1 for 24 weeks in GH deficient adults on (micro)vascular function, body composition and insulin resistance.

## Materials and Methods

### Study Design

This study presents data from a randomized, open-label, clinical trial conducted at one university hospital (VU University Medical Center, Amsterdam, The Netherlands) comparing decreasing and increasing GH dose for 24 weeks. The study investigates the efficacy and safety measures of GH replacement therapy before and after reaching low-normal and high-normal IGF-1 target levels (15). At entry subjects were receiving GH treatment according to general clinical practice (daily subcutaneous somatropin injections using automated pen systems manufactured by Pfizer Inc., Novo Nordisk Inc., and Eli Lilly and Co.). Subjects were selected based on having an IGF-1 concentration between −1 and 1 SDS (adjusted for age and gender) during GH replacement therapy. Randomization was done by a computer-generated random sequence and was stratified by gender. Subjects were randomized to receive either a decrease of their regular dose of GH treatment (IGF-1 target level of –2 to –1 SDS) (low dose=LD group), or an increase of their regular dose (IGF-1 target level of 1 to 2 SDS) (high dose=HD group), for 24 weeks. After 4 weeks the GH dose was adjusted when the target level of IGF-1 was not reached. At visit one (baseline) and visit two (after 24 weeks) blood samples were drawn and measurements performed to assess micro- and macrovascular function.

### Patients

The study group consisted of 32 adult patients with documented severe GH deficiency and more than one year of GH treatment, with an IGF-1 level between −1 and 1 SD score (SDS) for at least six months. Other pituitary hormone deficiencies had to be substituted when indicated and be stable for at least six months and during follow up. Severe GH deficiency was diagnosed prior to the study and defined according to the consensus guidelines of the GH Research Society for the diagnosis and treatment of adults with GH deficiency (16). In the Netherlands, the approval of GH treatment (and reimbursement of costs by the health insurer) was judged by an independent board of endocrinologists wanting to see two abnormal GH stimulation tests (mostly used: insulin-tolerance test and GHRH-arginin test) or one abnormal test or low IGF-1 in combination with panhypopituitarism or profound congenital GH deficiency. Patients were not eligible if they had a recent or current malignancy, craniopharyngioma as cause of hypopituitarism, were (planning to become) pregnant, or had a cardiovascular event within the last year before recruitment. Patients with prior Cushing’s disease or acromegaly were not excluded since an earlier study did not demonstrate significant interaction with the effect of GH treatment on cardiovascular mortality in GH deficient adults in The Netherlands (17). Patients were included after oral and written informed consent. The study protocol was approved by the Ethics Committee of the VU University Medical Center, Amsterdam. The study was performed according to Good Clinical Practice and the Declaration of Helsinki. This study is registered with ClinicalTrials.gov, number NCT01877512.

### Laboratory Investigations

Laboratory investigations included total IGF-1, and insulin and glucose to calculate insulin resistance by HOMA-IR. Blood samples were drawn after an overnight fast prior to every visit. Total IGF-1 was measured by a non-competitive (sandwich) chemiluminescence immunoassay (Liaison, DiaSorin S.p.A., Italy). The inter-assay coefficient of variation (CV) was 7.4%. Insulin was measured by an immunometric assay, Luminescence (Advia Centaur, Siemens Medical Solutions Diagnostics, USA). The inter-assay coefficient of variation (CV) was 8%.

### Microvascular Function

Endothelial function was assessed by microvascular measurements of the skin blood flow including endothelium (in)dependent vasodilatation and vasomotion. Endothelium-(in)dependent vasodilation of finger skin microcirculation was evaluated by measuring skin blood flow in perfusion units (PU) by a laser Doppler system (Periflux 4000, Perimed, Stockholm, Sweden) in combination with iontophoresis of acetylcholine (ACh) and sodium nitroprusside (SNP), respectively, as described previously (18). All measurements were performed in the fasting state, in the sitting position with the investigated hand at heart level in a temperature-controlled room. Skin temperature was registered continuously and was above 28°C at the start of all microvascular measurements. ACh (1% Miochol; Bournonville Pharma, Braine d’Alleud, Belgium) was delivered to the skin on the middle phalanx of the third finger using an anodal current, consisting of seven doses (0.1 mA for 20 s) with a 60 s interval between each dose. SNP (0.01%, Nipride; Roche, Woerden, The Netherlands) was delivered on the middle phalanx of the second finger using a cathodal current, consisting of seven doses (0.2 mA for 20 s) with a 90 s interval between each dose. In order to perform vasomotion analyses skin blood flow was measured during 30 min with a laser Doppler probe positioned at the dorsal side of the wrist of the arm. A bandpass filter with cut-off frequencies at 20 Hz and 20 kHz, and a time constant of 0.2 s, was selected. Wavelet analysis of the signals with a minimum of 30 min (with a sampling frequency of 32 Hz resulting in approximately 58.000 data points) in length was conducted to assess the frequency spectrum between 0.01 and 1.6 Hz. Wavelet analysis was performed using the wavelet toolbox in Matlab (7.8.0.347; The Mathworks, Inc., Natick, MA, USA), as described earlier (19). Scales are chosen for a resulting frequency range from 0.01 to 1.6 Hz which can be divided in five frequency intervals as described by Stefanovska et al. (20). The first three lower frequencies are locally generated; 0.01–0.02 Hz as endothelial activity, 0.02–0.06 Hz as neurogenic activity, and 0.06–0.15 Hz as myogenic response of the vascular smooth muscle cells (VSMC). The higher frequencies originate upstream and are: 0.15–0.4 Hz as respiratory function and 0.4–1.6 Hz as heart beat frequency. To eliminate edge effects, the first and last 2,000 samples were removed from the resulting wavelet transform. The relative amplitude was calculated for each of the five frequency bands by dividing the average amplitude within a band by the average amplitude of the entire spectrum. This normalization takes into account the variation in the signal strength between subjects and/or within subjects during an intervention (21).

### Macrovascular Hemodynamics and Vascular Stiffness

Blood pressure and heart rate were measured automated by Dinamap (PRO 100 V2), with a proper sized cuff, after 3 min of rest, three times with at least 1 min in between, where the two last measurements were averaged. Vascular stiffness was assessed by Pulse Wave Analysis, determining pulse wave velocity (PWV) and augmentation index (AIx) by a validated noninvasive automated device. The Sphygmocor Pulse Wave Velocity system uses applanation tonometry in conjunction with a three-lead ECG to take sequential measurements at two arterial sites. The timing of the onset of systole of the pressure waves were compared with the timing of the corresponding R waves on the ECG recording, with the same delay calculated by the software. PWV was calculated as the ratio of the distance traveled and the foot-to-foot time delay between pulse waves and expressed in meters per second. A high fidelity peripheral artery blood pressure waveform at the radial artery is used to calculate the AIx. The cardiac index (l/min/m^2^) at rest was determined by a non-invasive continuous hemodynamic monitoring system (Nexfin monitoring system, BMEYE B.V., Amsterdam, The Netherlands).

### Statistical Analyses

Categorical data were expressed as number (percentage) and continuous data as mean (SD), or as median (interquartile range (IQR)) for skewed variables. Parametric or non-parametric tests were used when appropriate. General Linear Model (GLM) for repeated measures was used for between-group differences for change over time. Skewed variables were transformed when needed. Adjustments for baseline value were conducted to account for regression to the mean for the different outcome measures. Moreover, at baseline the LD and HD groups differed with respect to childhood onset (CO) and adult onset (AO) GH deficiency, and this variable was therefore added as covariate to the final GLMs. Subsequently, linear regression analyses were performed to investigate whether the association of change in IGF-1 SDS with some domains of the vasomotion analysis remained when adjusting for relevant covariates. Data were examined by use of Pearson’s correlation. Two sided *P* values of 0.05 or less were considered significant. Statistical analyses were performed by the statistical software package IBM SPSS statistics 20.0 (SPSS Inc., Chicago, IL).

## Results

Between May 31, 2013, and April 11, 2014, we enrolled 32 patients. One subject withdrew after start of the study because of personal reasons. Another subject was excluded from the analyses due to the inability to reach the proper IGF-1 target level. The final analyses were conducted with 15 subjects in each group. **Table 1** shows the baseline characteristics of the groups. There were more subjects with CO GH deficiency in the LD group and consequently fewer patients with a history of pituitary surgery. This corresponds with the underlying diagnosis of GH deficiency being 50% congenital in the LD group (compared to 19% in the HD group) and 50% (treatment of) pituitary tumor in the HD group (compared to 25% in the LD group). Of all 13 pituitary tumors, six concerned a non-secreting adenoma, five a prolactinoma, and two an ACTH producing adenoma.

**Table 1:** Baseline characteristics of the low dose group in which the IGF-1 target level was between −2 and −1 standard deviation score (SDS), and the high dose group in which the IGF-1 target level was between 1 and 2 SDS.

	Low Dose		High Dose		*P value*
No. of patients	16		16		
Age, year	47.4	(10.8)	46.4	(9.3)	*0.80*
Sex, no. of females (%)	6	(37.5)	5	(31.2)	*0.71*
Onset of GHD, CO (%)	10	(62.5)	3	(18.8)	*0.01*
Cranial radiotherapy (%)	1	(6.2)	2	(12.5)	*1.00*
Pituitary surgery (%)	2	(12.5)	8	(50.0)	*0.02*
Isolated GHD (%)	4	(25)	4	(25)	*1.00*
TSH deficiency (%)	8	(50)	11	(68.8)	*0.28*
LH/FSH deficiency (%)	10	(62.5)	7	(43.8)	*0.29*
ACTH deficiency (%)	10	(62.5)	10	(62.5)	*1.00*
ADH deficiency (%)	0		4	(25)	*0.10*
Cardiovascular disease (%)	3	(18.8)	4	(25)	*1.00*
Diabetes mellitus (%)	3	(18.8)	0		*0.23*
Smoking (%)	4	(25)	2	(12.5)	*0.65*
GH dose, mg/day^a^	0.23	(0.36)	0.28	(0.30)	*0.93*
Duration GH treatment, year^a^	15.1	(17.9)	12.6	(12.7)	*0.09*

Values are mean (SD) unless stated otherwise.

a Median (IQR).

GHD, growth hormone deficiency; CO, childhood onset; GH, growth hormone.

**Figure 1:**
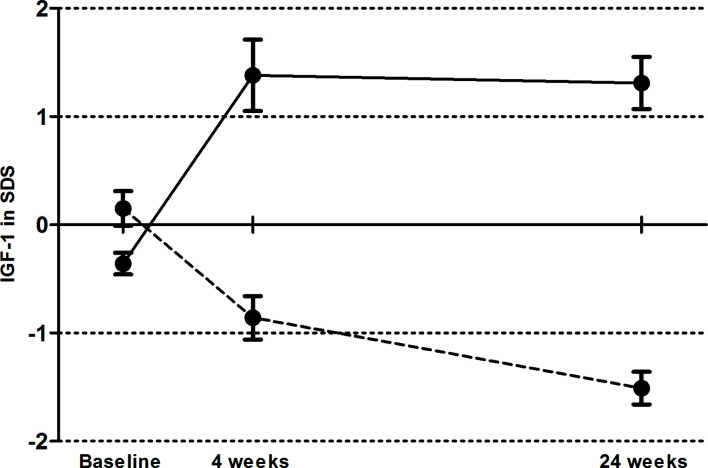
Mean serum total IGF-1 level in SD score (± SEM) at baseline, after four weeks of growth hormone (GH) treatment and at end of follow up in the low dose (dashed line) and high dose (solid line) group.

The median daily dose of GH was decreased from 0.25 (IQR 0.35) to 0.10 (IQR 0.15) mg/day (p<0.001) in the LD group and increased from 0.25 (IQR 0.30) to 0.50 (IQR 0.60) mg/day (p<0.001) in the HD group. The IGF-1 concentration decreased from 21.40 (SD 4.87) at baseline to 12.43 (SD 2.25) nmol/L (p<0.001) in the LD group after 24 weeks, and increased from 18.53 (SD 2.77) to 28.13 (SD 5.15) nmol/L (p<0.001) in the HD group (**Figure 1** shows the IGF-1 levels in SDS adjusted for age and gender).

**Figure 2:**
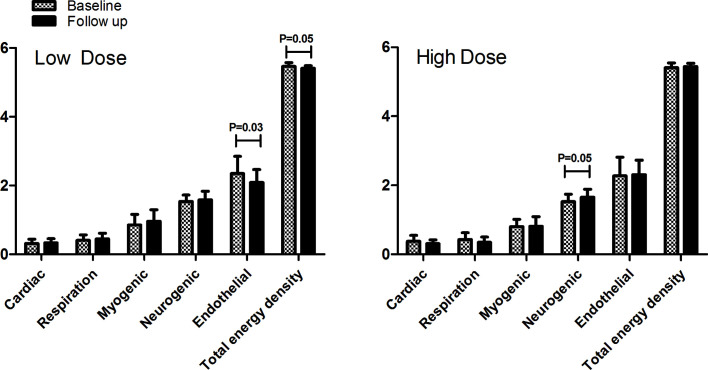
The contribution of different frequency intervals to microvascular vasomotion (expressed in energy density) by laser Doppler of skin blood flow in both treatment groups before and after 24 weeks. The frequency intervals are: 0.4–1.6 Hz = cardiac function, 0.15–0.4 Hz = respiratory function, 0.06–0.15 Hz = myogenic response of the vascular smooth muscle cells, 0.02–0.06 Hz = neurogenic activity, 0.01–0.02 Hz = endothelial activity.

The effects of increasing or decreasing IGF-1 levels on body composition, macrovascular hemodynamics and vascular stiffness and insulin resistance are presented in **Table 2**. In parallel to the favorable effect of increasing the IGF-1 level on waist circumference compared to decreasing the IGF-1 level (p=0.05), there was a significant difference in the effect on insulin resistance (p=0.03). Increasing IGF-1 by augmenting the GH dose in the HD group significantly increased insulin resistance compared to baseline (1.12 vs. 0.79, p=0.01), whereas no significant change was detected during decreased levels of IGF-1 (0.74 vs. 0.80, p=0.24). With respect to microvascular function, no (difference in) effect on endothelial-dependent, nor endothelial-independent, vasodilatation was found. Decreasing IGF-1 level significantly lowered the endothelial domain of vasomotion (p=0.03). Increasing IGF-1 level increased the contribution of the neurogenic domain (p=0.05) (**Figure 2**).

**Table 2:** The effect of increasing or decreasing IGF-1 level on measurements of body composition, insulin resistance, and macrovascular hemodynamics and vascular stiffness.

	Low Dose				High Dose				*P value for between group difference^a^*
	Baseline		Follow up		Baseline		Follow up		
BMI, kg/m^2^	28.2	(9.8)	28.2	(9.4)	28.8	(4.2)	28.4	(3.3)	*0.47*
Waist circumference, cm ^b^	97	(24)	99	(23)	105	(11)	102	(8)	*0.05*
Insulin resistance (HOMA-IR)^c^	0.80	(1.24)	0.74	(1.48)	0.79	(0.57)	1.12	(0.88)*	*0.03*
Fasting glucose, mmol/L	4.3	(0.7)	4.5	(1.2)	4.3	(0.9)	4.7	(0.6)	*0.54*
Insulin, pmol/L ^c^	43	(77)	40	(86)	42	(34)	62	(48)*	*0.01*
Total cholesterol, mmol/L	5.02	(1.01)	4.86	(0.75)	5.15	(0.98)	4.99	(1.02)	*0.90*
HDL cholesterol, mmol/L	1.51	(0.53)	1.54	(0.51)	1.47	(0.40)	1.50	(0.43)	*0.63*
LDL cholesterol, mmol/L	3.04	(0.88)	2.86	(0.56)	3.10	(0.94)	2.92	(0.79)	*0.93*
Triglycerides, mmol/L	1.03	(0.38)	1.03	(0.40)	1.27	(0.59)	1.33	(0.45)	*0.65*
Free fatty acid, mmol/L	0.42	(0.18)	0.42	(0.19)	0.45	(0.22)	0.51	(0.22)	*0.58*
Systolic blood pressure, mmHg	129	(18)	127	(12)	126	(14)	124	(16)	*0.71*
Diastolic blood pressure, mmHg	80	(8)	78	(8)	77	(9)	74	(10)	*0.42*
Heart rate, beats/min	68	(8)	63	(8)*	59	(8)	61	(9)	*0.54*
Cardiac index, liter/min/m^2^	3.0	(0.5)	3.0	(0.7)	2.7	(0.5)	2.9	(0.4)	*0.36*
Pulse Wave Velocity, m/s	7.2	(1.0)	7.2	(0.7)	7.1	(1.2)	7.1	(1.5)	*0.76*
Augmentation index	24	(18)	25	(17)	20	(15)	17	(11)	*0.17*

Values are mean (SD) unless stated otherwise.

^a^The effect in both groups (delta) are compared and adjusted for baseline value and onset of GHD, ^b^Waist circumference reference value men <102 cm, women <88 cm, ^c^Median (IQR), *P value < 0.05 for change from baseline.

**Figure 3:**
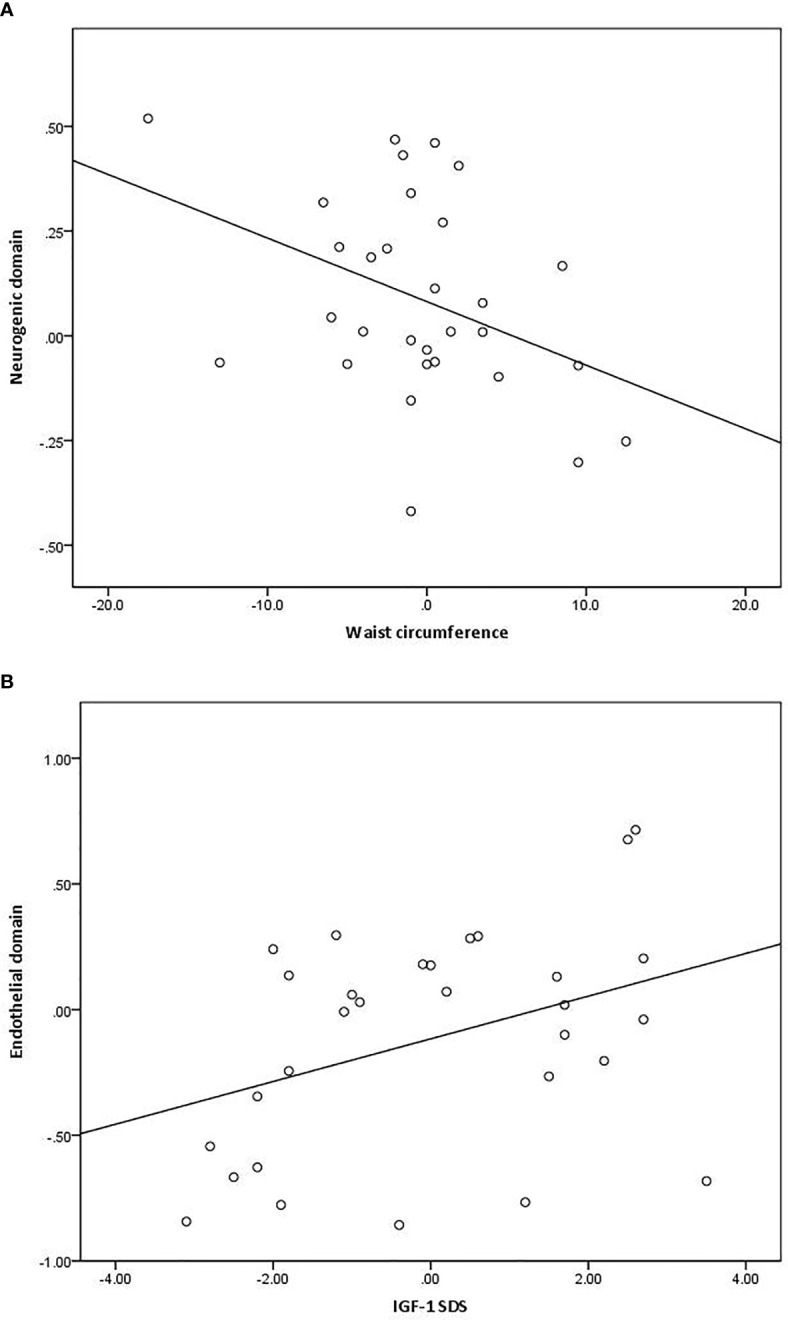
Associations between change in the activity of the neurogenic vasomotion domain and waist circumference [**(A)**; r −0.39, p<0.05] and the change in the activity endothelial vasomotion domain and IGF-1 standard deviation score (SDS) [**(B)**; r 0.38, p<0.05].

Correlation analyses (**Figure 3**) demonstrated that the change in waist circumference was inversely correlated with the change in the neurogenic vasomotion domain (r −0.39, p<0.05), but not with change in IGF-1 SDS or HOMA-IR. In addition, the change in IGF-1 SDS was positively correlated with the change in the endothelial vasomotion domain (r 0.38, p<0.05), but not with changes in waist circumference or HOMA-IR. Subsequently, these associations and possible confounders were explored in the regression models presented in **Table 3**.

**Table 3:** Regression analysis for change in IGF-1 standard deviation score (SDS) and the change in neurogenic and endothelial vasomotion domains and adjusted for change in waist circumference and/or insulin resistance.

	Neurogenic domain			Endothelial domain		
	B	95% CI	P value	B	95% CI	P value
**Model 1**						
IGF-1 SDS	0.032	(−0.014–0.078)	0.16	0.085	(0.004–0.166)	0.04
**Model 2**						
IGF-1 SDS	0.015	(−0.033–0.064)	0.52	0.073	(−0.017–0.162)	0.11
Waist circumference	-0.013	(−0.029–0.002)	0.09	−0.009	(−0.037–0.019)	0.50
**Model 3**						
IGF-1 SDS	0.019	(−0.032–0.071)	0.45	0.078	(−0.014–0.171)	0.10
Insulin resistance	0.112	(−0.087–0.311)	0.26	0.058	(−0.299–0.416)	0.74
**Model 4**						
IGF-1 SDS	0.001	(−0.053–0.054)	0.97	0.065	(−0.036–0.166)	0.20
Waist circumference	−0.014	(−0.029–0.002)	0.08	−0.010	(−0.038–0.019)	0.50
Insulin resistance	0.119	(−0.073–0.310)	0.21	0.063	(−0.299–0.425)	0.72

## Discussion

This exploratory study on the possible mechanisms linking IGF-1 and CVD investigated the effect on (micro)vascular function, body composition and insulin resistance of changing the IGF-1 concentration to low- or high-normal levels during GH treatment in GH deficient adults. The most clear finding was that increasing the GH dose to a high-normal IGF-1 level led to a significant increase in insulin resistance, but a reduction in waist circumference. Moreover, although the overall effect on (micro)vascular function was limited, both the neurogenic and endothelial vasomotion domain were affected by a change in the GH dose. Interestingly, insulin resistance and (central) obesity have been demonstrated to be associated with a decreased activity of the neurogenic and endothelial vasomotion domain (22). In the present study, however, the changes in insulin resistance and the vasomotion domains were disconcordant, i.e. insulin resistance became worse, whereas the contribution of the neurogenic and endothelial vasomotion domains increased after increasing the GH dose to a high-normal IGF-1 level. The changes in vasomotion seem, in part, to parallel the changes in waist circumference.

Vasomotion is the rhythmic change in vascular diameter which is thought to influence capillary density and capillary exchange of substances between blood and tissues (23). As already mentioned IGF and insulin receptors can be detected on the microvascular endothelium, and therefore IGF and insulin should in theory be able to influence microvascular vasomotion. Indeed, insulin has been shown to alter arteriolar vasomotion with a resultant increase in the capillary exchange surface (14, 24, 25). Systemic hyperinsulinemia affects vasomotion by increasing neurogenic and endothelial activity in skin, and the change in the neurogenic vasomotion domain is directly associated with the increase in capillary density during hyperinsulinemia (14, 25). Moreover, in obese, insulin-resistant subjects, the contribution of the neurogenic and endothelial vasomotion domains is impaired (22). Data on the effects of GH on vasomotion are presently lacking, but the finding that GH replacement therapy is able to increase capillary density in a similar fashion as insulin, suggests that IGF-1 may influence vasomotion. Indeed, in the present study, increasing IGF-1 level leading to significantly more insulin resistance but a lower waist circumference, resulted in more neurogenic activity, whereas decreasing IGF-1 levels resulted in less endothelial activity in the vasomotion analysis. However, increasing IGF-1 levels did result in a reduction in waist circumference, which could have had a favorable effect on the microcirculation. These results are in line with previous studies investigating microvascular vasomotion. De Jongh et al. found that the contribution of the frequency spectrum of the neurogenic activity to vasomotion was lower in obese compared to lean women (22). De Boer et al. demonstrated an inverse association of body mass index and trunk fat with the neurogenic vasomotion domain in a different cohort (21). However, these studies were both cross-sectional. This study now demonstrates that by changing waist circumference (by changing IGF-1 level) the neurogenic domain of vasomotion is affected, which strengthens this finding. Next to the change in neurogenic activity, decrease in IGF-1 level in our study led to a decreased contribution of the endothelial activity to the vasomotion, which seemed to be independent of change in waist circumference or insulin resistance. Consequently, this appears to be a direct effect of low IGF-1, perhaps due to the decreased formation of NO (26). Studies on the effect of IGF-1 on endothelial function are scarce. Endothelial cells have high-affinity IGF-1 binding sites and IGF-1 stimulates NO formation by endothelial cells and VSMCs (27). Christ et al. demonstrated in patients with GH deficiency that GH treatment had a beneficial effect on endothelial function (measured by using venous occlusion plethysmography before and after infusion of ACh and of SNP) mediated by endothelium-dependent NO production and/or increase in sensitivity of VSMC to NO (28). One might have expected also an improvement of endothelial function and arterial stiffness in the present study due to the effect of raising NO formation by increasing IGF-1 levels. The lack of effect could be due to sample size and limited follow up time in which the IGF-1 levels were adjusted in a relative small width. Rossi et al. (23) also suggest that skin vasomotion investigation is a more sensitive evaluation of microvascular endothelial function than skin blood flow measurements, explaining that in our study the effect on endothelial function was first demonstrated in the vasomotion measurements after change of GH dose.

This is one of the first studies to explore possible mechanisms for the association of IGF-1 levels within the normal range with cardiovascular risk factors in GH treated GH deficient adults. As mentioned above, a limitation of the study is the overlapping effects of both higher GH doses and higher IGF-1 level which could have influenced the results, for instance with respect to dose-dependent effect of GH on insulin resistance (29). Another factor to take into account when interpreting the results is the total number of statistical tests performed in a relatively small sample. Some of the findings could have been due to chance alone. However, most changes were in the expected direction and a larger sample size or prolonged duration of the intervention with proper adjustments for multiple testing could be expected to demonstrate similar results.

In conclusion, in this exploratory study to elucidate possible mechanism underlying the U-shaped relationship of IGF-1 with CVD, we demonstrated that higher IGF-1 levels are beneficial for body composition but seem to be detrimental with respect to insulin resistance. The contribution of the neurogenic vasomotion domain increased in parallel, and could be explained by the favorable change in waist circumference. It remains to be seen whether the effects on the neurogenic vasomotion domain are indeed beneficial for capillary perfusion and cardiovascular homeostasis, and therefore can be considered a measure of optimal IGF-1 levels. Since the really long-term effect of high-normal IGF-1 target levels during GH treatment remain to be investigated, and also the known suggested association with tumor progression, no clear recommendation on dosing strategy can be given at this moment.

## Data Availability Statement

The raw data supporting the conclusions of this article will be made available by the authors, without undue reservation.

## Ethics Statement

The studies involving human participants were reviewed and approved by Ethics Committee of the VU University Medical Center, Amsterdam. The patients/participants provided their written informed consent to participate in this study.

## Author Contributions

CV and MD were responsible for design, execution, analysis of the study, and writing of the manuscript. RM, PL, MK, and ES thoroughly assisted in the design of the study, analysis and writing of the manuscript. All authors contributed to the article and approved the submitted version.

## Funding

CV is supported by an AGIKO grant of The Netherlands Organisation for Health Research and Development (ZonMw) (grant number: 92003591). This work was partly supported by an investigator-initiated grant from Pfizer bv.

## Conflict of Interest

The authors declare that the research was conducted in the absence of any commercial or financial relationships that could be construed as a potential conflict of interest.
